# The Saline Waters of Leamington

**Published:** 1885-12

**Authors:** 


*The Saline Waters of Leamington.* By F. W. SMITH, M.D. Second Edition. Pp. 96. London: H. K. LEWIS. 1885.

The second edition of this little work, which we have already favourably commented upon, is enriched by the insertion of three separate analyses of the Leamington waters by distinguished chemists, and by other additions which will increase its value. It is a handy, unassuming, and trustworthy guide to one of the most popular of our English spas.

